# Limited Effect of Chronic Valproic Acid Treatment in a Mouse Model of Machado-Joseph Disease

**DOI:** 10.1371/journal.pone.0141610

**Published:** 2015-10-27

**Authors:** Sofia Esteves, Sara Duarte-Silva, Luana Naia, Andreia Neves-Carvalho, Andreia Teixeira-Castro, Ana Cristina Rego, Anabela Silva-Fernandes, Patrícia Maciel

**Affiliations:** 1 Life and Health Sciences Research Institute (ICVS), School of Health Sciences, University of Minho, 4710–057 Braga, Portugal; 2 ICVS/3Bs—PT Government Associate Laboratory, Braga/Guimarães, Portugal; 3 CNC-Center for Neuroscience and Cell Biology, University of Coimbra, Coimbra, Portugal; 4 Faculty of Medicine, University of Coimbra, Coimbra, Portugal; University of Pennsylvania Perelman School of Medicine, UNITED STATES

## Abstract

Machado-Joseph disease (MJD) is an inherited neurodegenerative disease, caused by a CAG repeat expansion within the coding region of *ATXN3* gene, and which currently lacks effective treatment. In this work we tested the therapeutic efficacy of chronic treatment with valproic acid (VPA) (200mg/kg), a compound with known neuroprotection activity, and previously shown to be effective in cell, fly and nematode models of MJD. We show that chronic VPA treatment in the CMVMJD135 mouse model had limited effects in the motor deficits of these mice, seen mostly at late stages in the motor swimming, beam walk, rotarod and spontaneous locomotor activity tests, and did not modify the ATXN3 inclusion load and astrogliosis in affected brain regions. However, VPA chronic treatment was able to increase GRP78 protein levels at 30 weeks of age, one of its known neuroprotective effects, confirming target engagement. In spite of limited results, the use of another dosage of VPA or of VPA in a combined therapy with molecules targeting other pathways, cannot be excluded as potential strategies for MJD therapeutics.

## Introduction

Polyglutamine (PolyQ) diseases are neurodegenerative disorders caused by an expansion of trinucleotide CAG repeats within the coding region of specific genes [1]. This group of disorders includes spinal bulbar muscular atrophy (SBMA), Huntington’s disease (HD), Dentatorubral-Pallidoluysian atrophy (DRPLA), and six types of spinocerebellar ataxias (SCA’s) [2]. Machado-Joseph disease (MJD) or Spinocerebellar Ataxia type 3 (SCA3) is the most common dominantly inherited SCA worldwide and is caused by the expansion of a polyQ tract in the C-terminus of the *ATXN3* gene product [3]. Both the normal and expanded ataxin-3 (ATXN3) proteins are expressed ubiquitously, although the neurodegeneration in MJD is limited to some brain regions, mainly in cerebellum, brainstem and spinal cord [4]. The symptoms include ataxia, progressive external ophthalmoplegia, pyramidal and extrapyramidal signs, peripheral amyotrophies, intention fasciculation-like movements of facial and lingual muscles, rigidity, and bulging eyes [5–7]. The pathological hallmark of the disease is the presence of neuronal intranuclear inclusions (NIIs) of aggregation-prone expanded ATXN3 in the patients' brain, being the pathogenic relevance of these aggregates still unclear [8–11].

Despite the recent efforts towards the understanding of the pathogenesis of this disorder, the molecular pathways that ultimately lead to neuronal demise remain mostly unknown and no effective treatments are yet available for MJD, as for other polyQ diseases. Nevertheless, there seem to be common pathways between all polyQ diseases that were shown to be altered, which could be explored in the development of therapeutics, related to transcriptional dysregulation, mitochondrial dysfunction, oxidative stress, Ubiquitine Proteasome System (UPS) impairment, excitotoxicity, DNA damage and activation of apoptotic pathways [12].

Nevertheless, the translation of candidate therapies to clinical trials is a very long process due to uncertainty for human safety and has not improved significantly in the last years. In this context, drug re-purposing strategies, which relies on finding new uses for existing FDA-approved compounds, has been gaining attractiveness due to the faster translation to the clinic, with predictably less safety issues.

VPA is an FDA-approved compound that has been used over the years as an anticonvulsant and mood-stabilizing drug in the treatment of epilepsy, bipolar disorder and migraine [13], with a relatively safe profile in clinical use. In the last years, a growing body of evidence indicates that VPA holds promise in treating other neurodegenerative diseases due to its diverse mechanisms of action. Its pharmacological effects comprise a range of mechanisms, including inhibition of histone deacetylases, increased gamma-aminobutyric acid (GABA)-ergic transmission, reduced release and/or effects of excitatory amino acids, blockade of voltage-gated sodium channels and modulation of dopaminergic and serotoninergic transmission [14]. VPA treatment is also known to produce changes in the expression of multiple genes, involved in transcription regulation, cell survival, ion homeostasis, cytoskeletal modifications, signal transduction, endoplasmic reticulum stress and longevity [13,15] This drug has been shown to delay the disease onset, to reduce neurological deficits and/or to prolong survival in several models of neurodegenerative diseases, including HD, SBMA and Amyotrophic Lateral Sclerosis (ALS) [16–18]. In MJD, VPA was reported to alleviate neurodegeneration in a *Drosophila* model of the disease [19] and to attenuate mutant ATXN3-induced cell toxicity in a human neuronal cell model [20]. Moreover, we have previously shown a significant reduction of mutant ATXN3 aggregation and neurological dysfunction in a *C*. *elegans* model of MJD upon VPA treatment through the protective role of the transcription factor DAF-16, supporting a role in protection against proteotoxicity related to aging and cell survival [21]. However, its therapeutic efficacy is still not demonstrated in a mammalian model of MJD. The goal of this work was to test the therapeutic efficacy of chronic VPA treatment in a mouse model of MJD, CMVMJD135 [22]. Our results show that chronic VPA treatment at the dosage used in this pre-clinical trial, lead to very limited and transient phenotypic effects in the CMVMJD135 mouse model, and did not change the ATXN3 inclusion load neither astrogliosis in affected brain regions.

## Results

### Effect of VPA acute treatment in histone acetylation and neuroprotective molecules in the cerebellum

**Fig 1:**
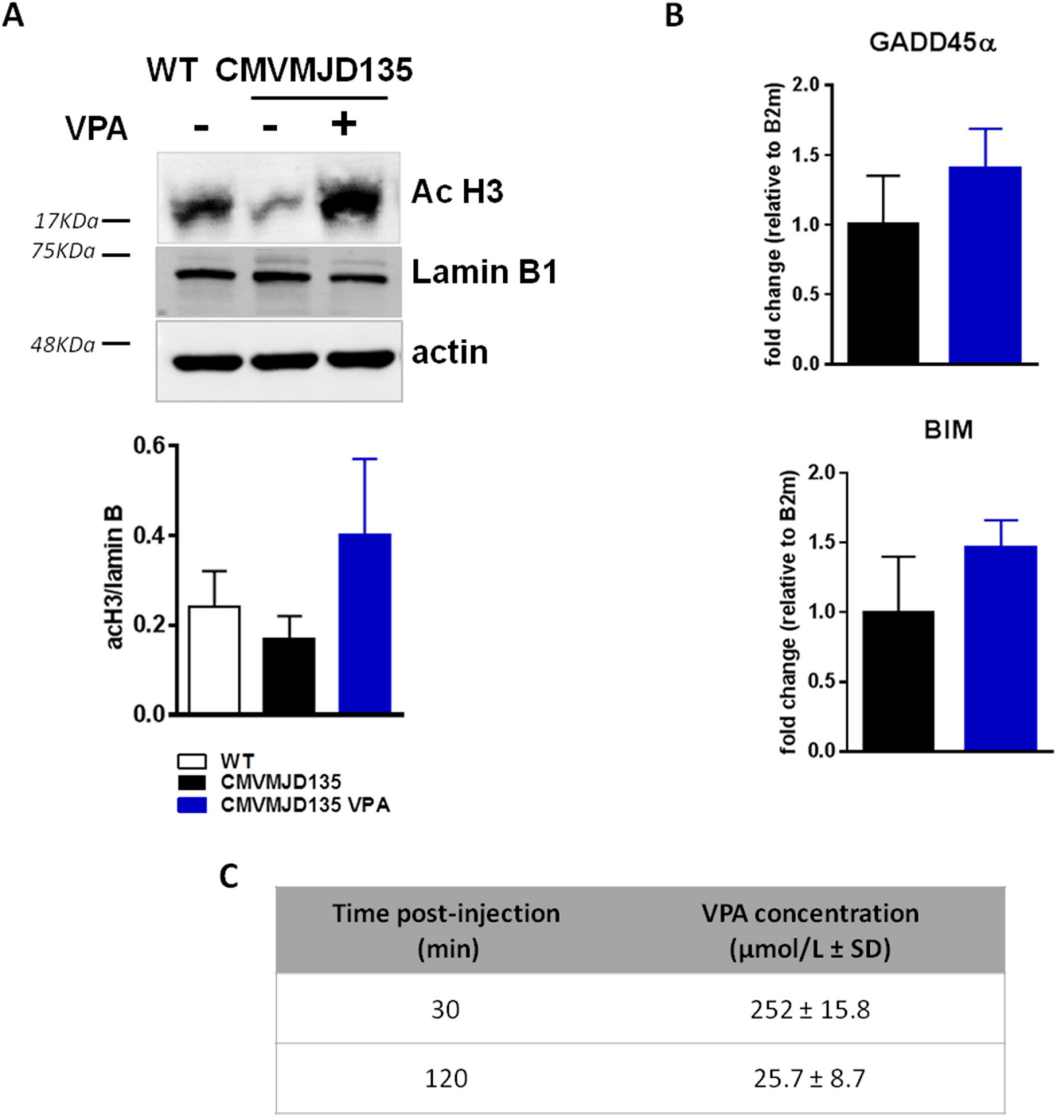
VPA acute treatment effects in cerebellum of CMVMJD135 mouse model. (A) A trend towards an increase in the H3 histone acetylation upon VPA acute treatment; (B) a trend towards an increase in GADD45α and BIM mRNA levels upon VPA acute treatment and (C) VPA concentration in the plasma after 30 and 120 minutes post-injection. Bars represent the mean ± SEM (n = 4 males for each group), One-Way ANOVA.

Although CMVMJD135 mouse model do not present a general hypoacetylation of histones in specific brain regions, we measured the H3 acetylation and we observed a trend towards an increase in H3 acetylation levels upon 5 days of VPA acute treatment (Fig 1A)

This result did not present statistical difference due to high variability between samples, however, a trend was observed to an hyperacetylation of histone H3 in the cerebellum upon VPA treatment, when normalized to the nuclear protein Lamin B1. Furthermore, and considering our previous results in *C*.*elegans* model of MJD upon VPA treatment, we also assessed the mRNA levels of GADD45α and BIM, two genes related to stress resistance and apoptosis regulation, respectively [23–26], in the cerebellum of CMVMJD135 mice, where a trend towards an increase upon VPA acute treatment was also observed (Fig 1B).

Plasma VPA concentration was also assessed after 30 and 120 minutes post-injection. An average of 252 ± 15.8 μmol/L after 30 minutes was observed in VPA-treated animals while a 25.7 ± 8.7 μmol/L concentration was detected after 120 minutes post-injection (Fig 1C).

### VPA treatment had limited effects in neurological deficits and decreased the body weight gain in CMVMJD135 mice

**Fig 2:**
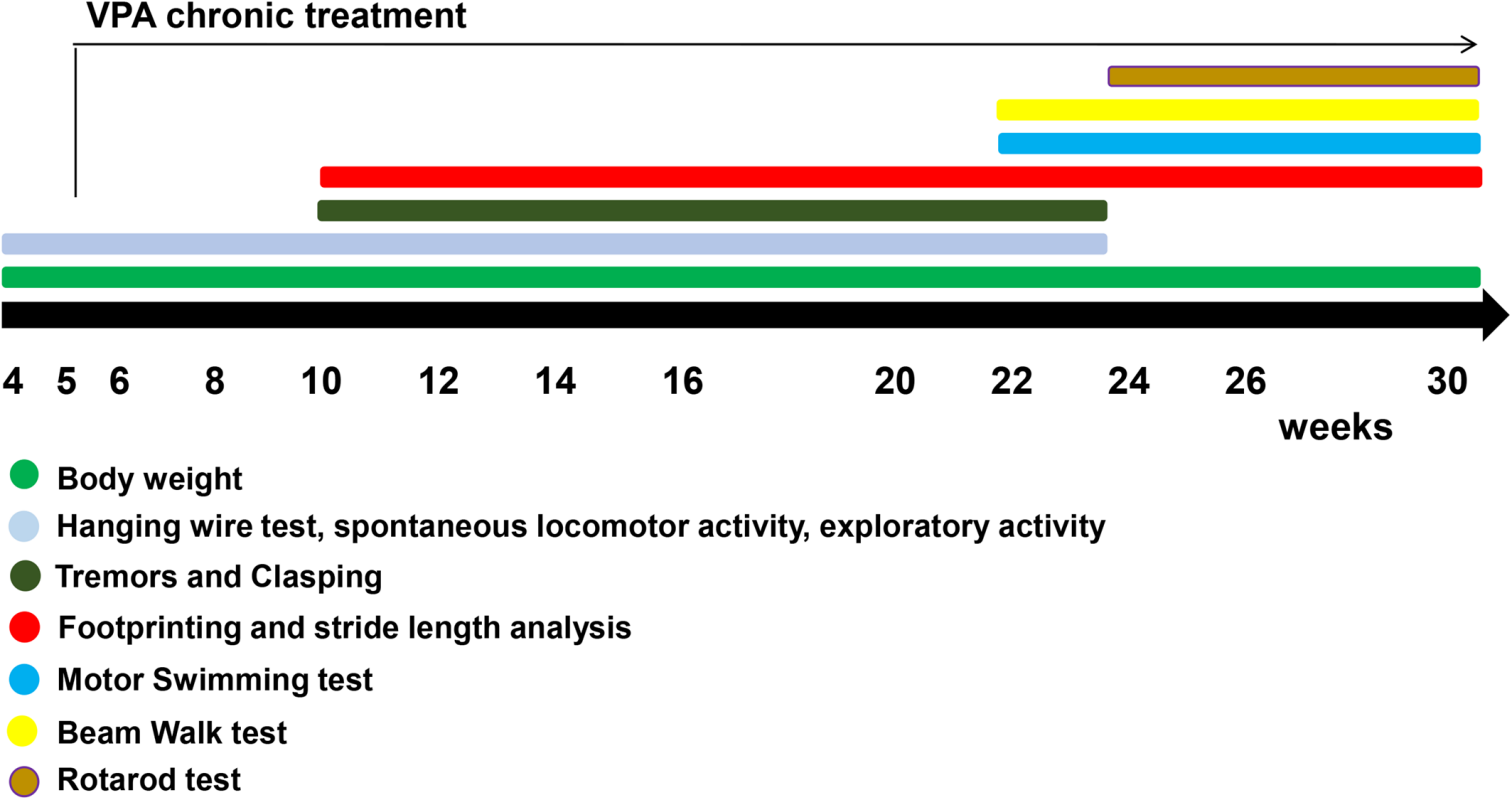
Schematic representation of the VPA pre-clinical therapeutic trial.

Chronic VPA treatment was initiated at 5 weeks of age, with a dosage of 200mg/kg, for 5 consecutive days each week, until 30 weeks of age. A battery of neurological and motor coordination tests was performed since 4 weeks of age until 30 weeks of age (Fig 2).

**Fig 3:**
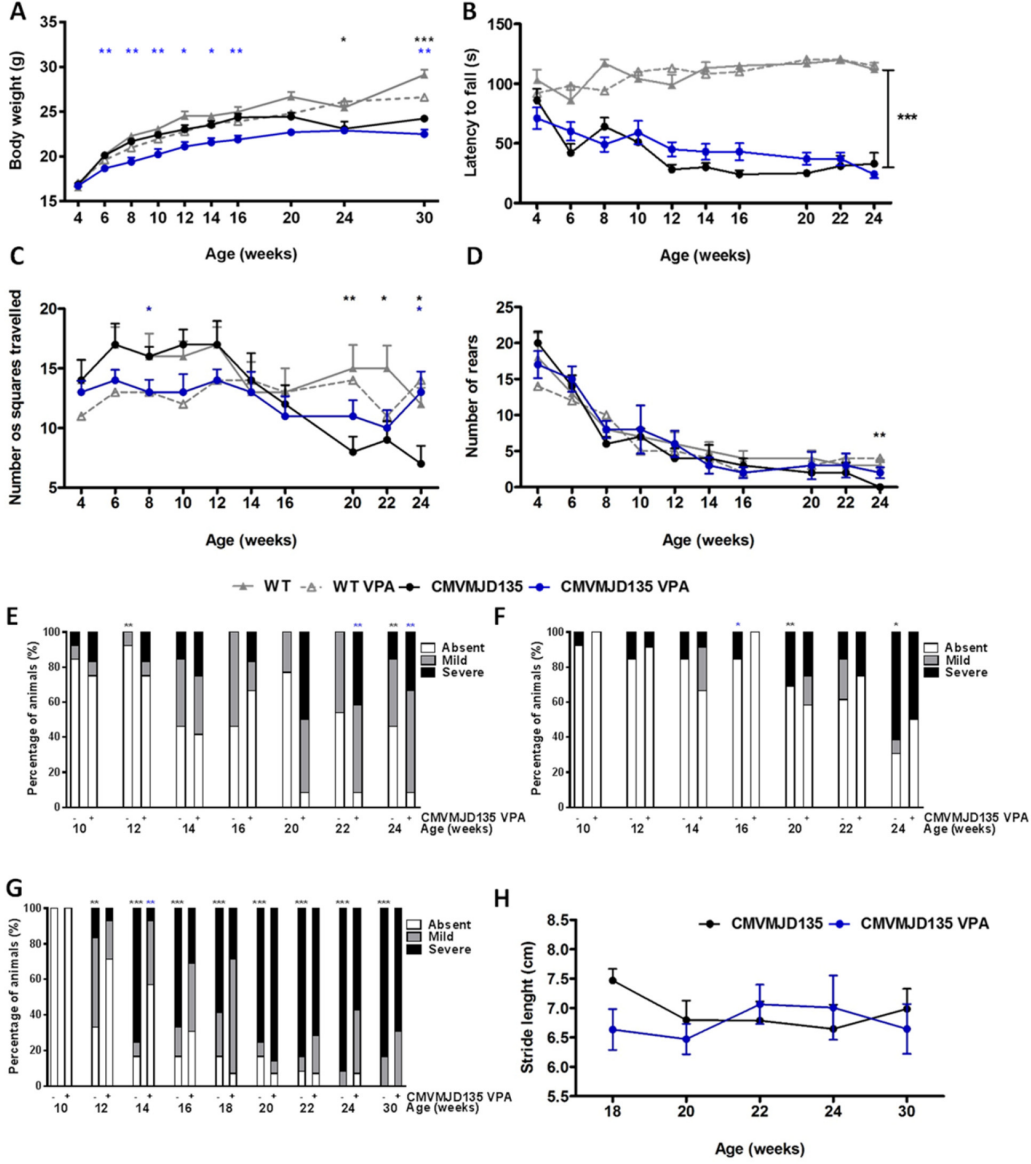
Minor effects in neurological deficits and body weight gain presented by CMVMJD135 mice upon VPA treatment. (A) Decreased body weight gain in CMVMJD135 VPA-treated animals compared to CMVMJD135 vehicle-treated animals; (B) no improvement of VPA-treated animals in grip strength as assessed through the hanging wire test; (C) Improvement in spontaneous locomotor activity at 24 weeks of age; (D,E,F) No improvement in spontaneous activity (vertical movement), in tremors and limb clasping, respectively, (G) transient improvement in footdragging severity at 14 weeks, and (H) tendency toward an improvement in stride length at 22 and 24 weeks of age. Bars represent the mean ± SEM (WT veh, n = 10; WT VPA, n = 15; CMVMJD135 vehicle, n = 10; CMVMJD135 VPA, n = 13), * represent p<0.05, ** represent p<0.01 and *** represent p<0.001, black asterisks represents the difference between WT and CMVMJD135, blue asterisks represents the difference between non-treated and VPA-treated CMVMJD135 (Repeated-measures ANOVA, Tukey correction for continuous variables, One-Way ANOVA for differences between groups in specific ages of the continuous variables and Chi-square Fisher’s exact test for categorical non-continuous variables).

No differences were found between WT and CMVMJD135 mice at 4 weeks of age before the beginning of the injections. CMVMJD135 mice start showing less body weight gain at 16 weeks of age, being statistically different from age-matched WT littermates at 24 weeks of age (Fig 3A). VPA treatment significantly reduced the already diminished body weight gain of the transgenic animals since very early in this trial (Fig 3A) suggesting some toxicity to these animals.

Other than reduced weight gain, no apparent clinical symptoms indicative of significant health impact were observed during long-term VPA treatment of WT and transgenic animals, and no more than 20% of their total body weight was lost at any instance. The first sign of neurological disease in the CMVMJD135 mouse model is the presence of muscular grip strength abnormalities at 6 weeks of age, given by the significant decrease in the latency to fall off in the hanging wire test [22]. VPA treatment did not alter the progression of the CMVMJD135 animals in the hanging wire, demonstrating an absence of effect in muscle strength and/or fine motor coordination of the paws (Fig 3B). Spontaneous locomotor activity of transgenic animals, given by the number of squares travelled in the arena, was markedly increased upon VPA treatment at 24 weeks of age (Fig 3C). However, spontaneous vertical exploratory activity, tremors and clasping were not improved by VPA treatment (Fig 3D, 3E and 3F).

Gait abnormalities were assessed qualitatively by the analysis of the footprint pattern. CMVMJD135 mice model presented foot dragging already at 12 weeks progressing through age. VPA was able to decrease the severity of this phenotype only at 14 weeks (Fig 3G), while no effect was observed at more advanced stages of the trial. In addition, stride length was also measured and although not significant, a trend towards and improvement in transgenic animals upon VPA treatment was observed at 22 and 24 weeks of age (Fig 3H).

### Long-term VPA treatment led to limited improvement in balance and motor coordination at later disease stages

Since chronic VPA treatment induced a decrease in body weight of transgenic animals, behavior performance in the motor and balance coordination tests was normalized to body weight. Results of behavioral tests without normalization to body weight are included in supplementary data (S1 Fig).

**Fig 4:**
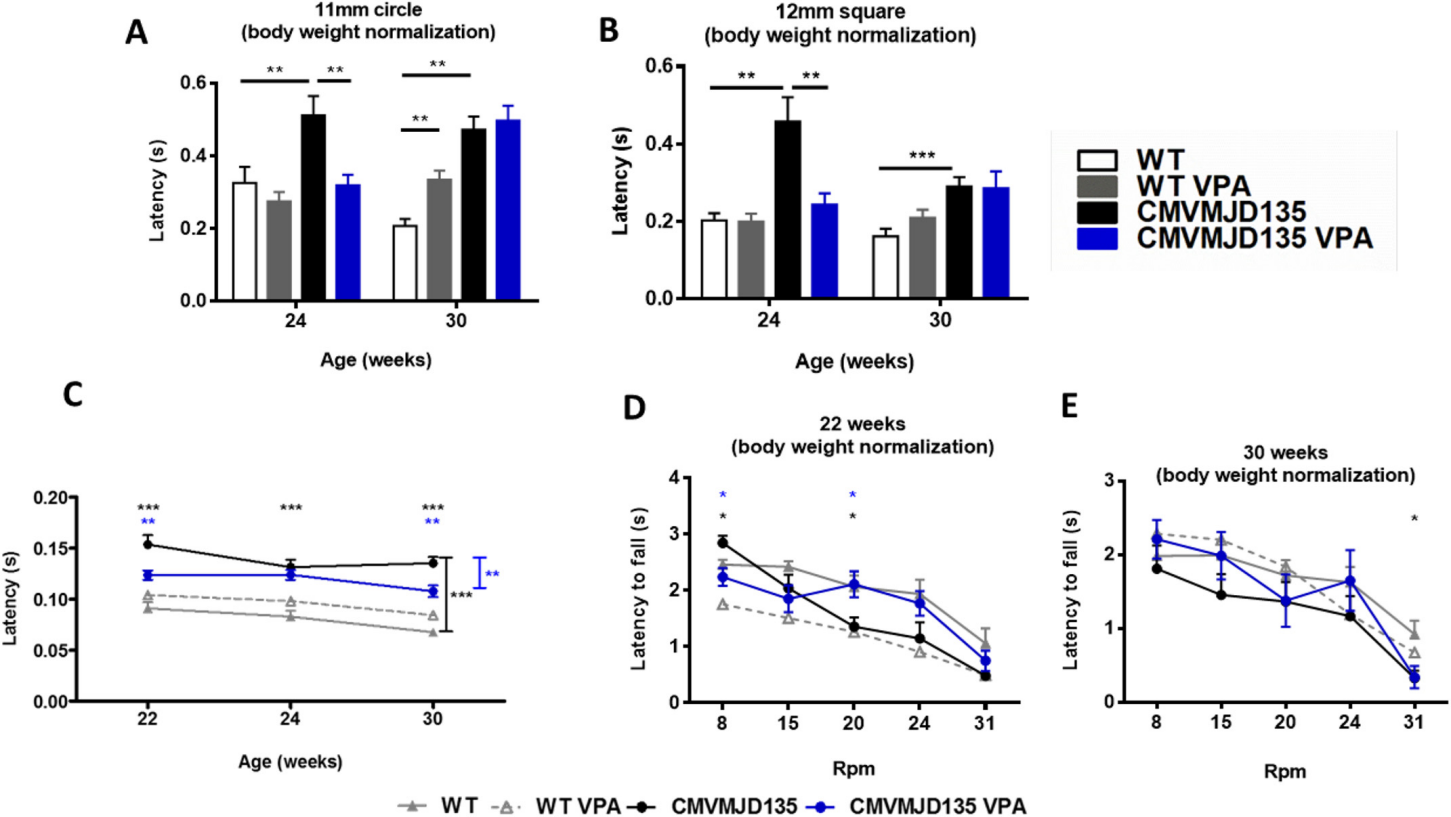
Balance and motor coordination performance normalized for animal body weight. (A,B) No differences were observed between non-normalized and normalized performance for body weight in balance beam walk test; (C) improvement motor swimming test at 22 and 30 weeks of age and (D) improvement at 22 weeks of in 8 and 20 rpm of Rotarod test. Bars represent the mean ± SEM (WT veh, n = 10; WT VPA, n = 15, CMVMJD135 vehicle, n = 10; CMVMJD135 VPA, n = 13), * represent p<0.05, ** represent p<0.01 and *** represent p<0.001, black asterisks represents the difference between WT and CMVMJD135, blue asterisks represents the difference between non-treated and VPA-treated CMVMJD135, (Repeated-measures ANOVA, Tukey correction for continuous variables, One-Way ANOVA for differences between groups in specific ages of the continuous variables and Mann-Whitney U test for continuous variables without normal distribution (Rotarod).

VPA treatment ameliorated balance and motor coordination of CMVMJD135 mice at 24 weeks of age, after 20 weeks of daily treatment, as assessed by the time taken to cross the 11 mm circle and 12 mm beams in the balance beam walk test. However, this improvement was not maintained at 30 weeks of age (Fig 4A and 4B).

In the motor swimming test, VPA-treated CMVMJD135 mice also had a better performance later in life (22 and 30 weeks of age) when compared to vehicle-treated mice (Fig 4C). Other motor deficits observed in CMVMJD135 mice, namely the loss of motor coordination observed in the Rotarod test, were alleviated by VPA treatment at 22 weeks of age for 8 and 20 rpm, but not maintained at 30 weeks of age. (Fig 4D and 4E).

### VPA treatment did not change the ataxin-3 inclusion load and astrogliosis in specific brain regions of CMVMJD135 mice

**Fig 5:**
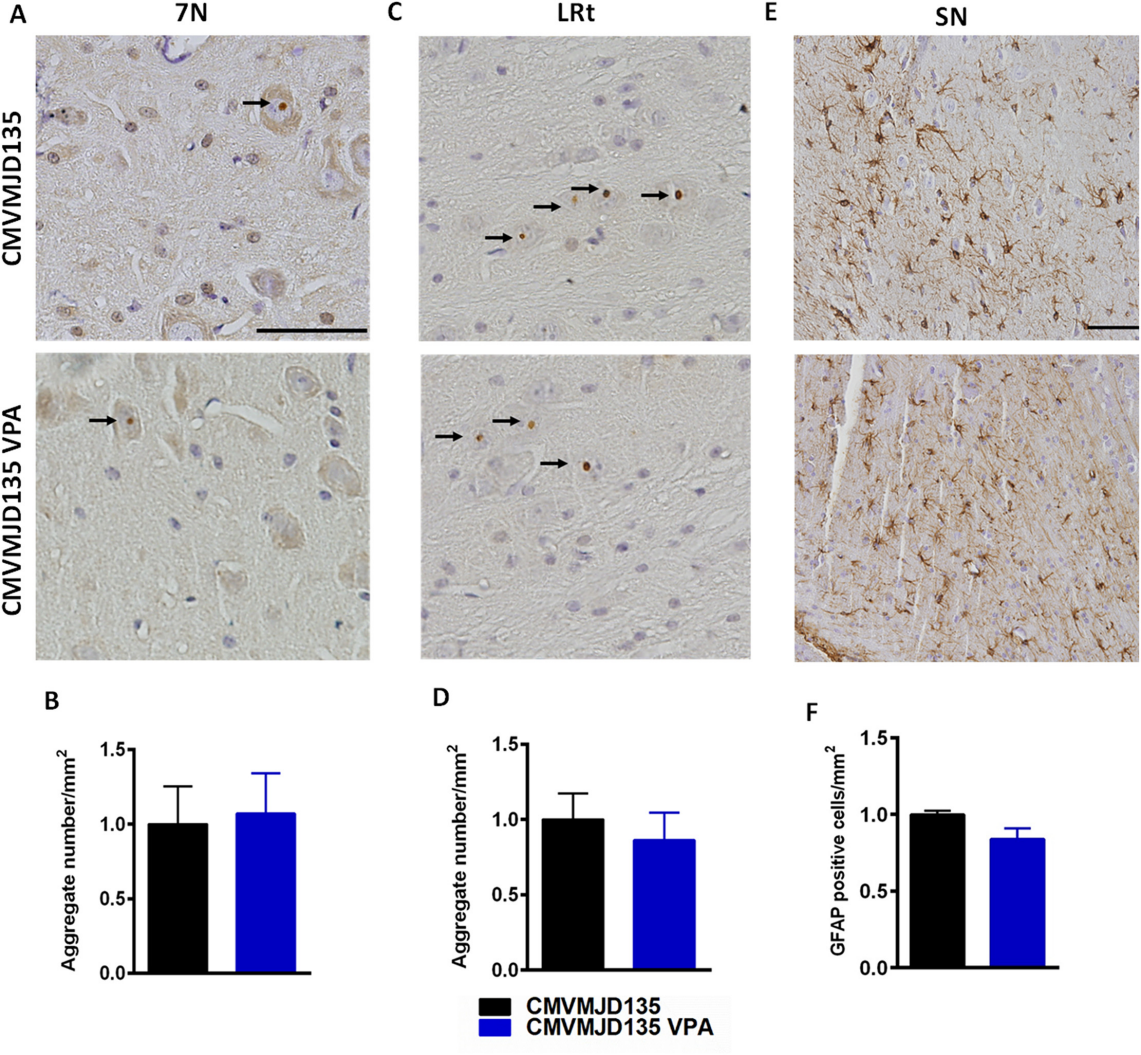
Immnuhistochemistry and quantification of ATXN3 neuronal inclusions and astrogliosis of VPA-treated and non-treated CMVMJD135. No differences in nuclear ATXN3 inclusion load were observed between groups in (A,B) 7N and (C,D) LRt brain regions. No differences in astrogliosis in substantia nigra (SN) between VPA-treated and non-treated transgenic animals (E,F). Scale bar of ATXN3 figures, 20 μm; Scale bar of GFAP figure, 200 μm.Bars represent the mean ± SEM (n = 4 for each group), One-Way ANOVA.

At the pathological level, CMVMJD135 mice show presence of ATXN3 NIIs in the nucleus of cells in different regions of the CNS including the pontine nuclei, reticulotegmental nucleus of the pons, spinal cord neurons, facial motor nuclei, anterior olfactory nuclei, ventral tenia tecta, inferior olive, dentate nuclei, locus coeruleus, cuneate nuclei and lateral reticular nuclei [22]. The analysis of brain tissue of VPA-treated and non-treated CMVMJD135 by immunohistochemistry of ATXN3 in facial motor nuclei (7N) and lateral reticular nuclei (LRt), did not reveal significant differences between groups (Fig 5A–5D).

Astrogliosis observed in substantia nigra of the CMVMJD135 mouse model, was also not mitigated upon VPA treatment (Fig 5E and 5F).

### Chronic VPA treatment increases GRP78 protein levels in cerebellum of CMVMJD135 mice

**Fig 6:**
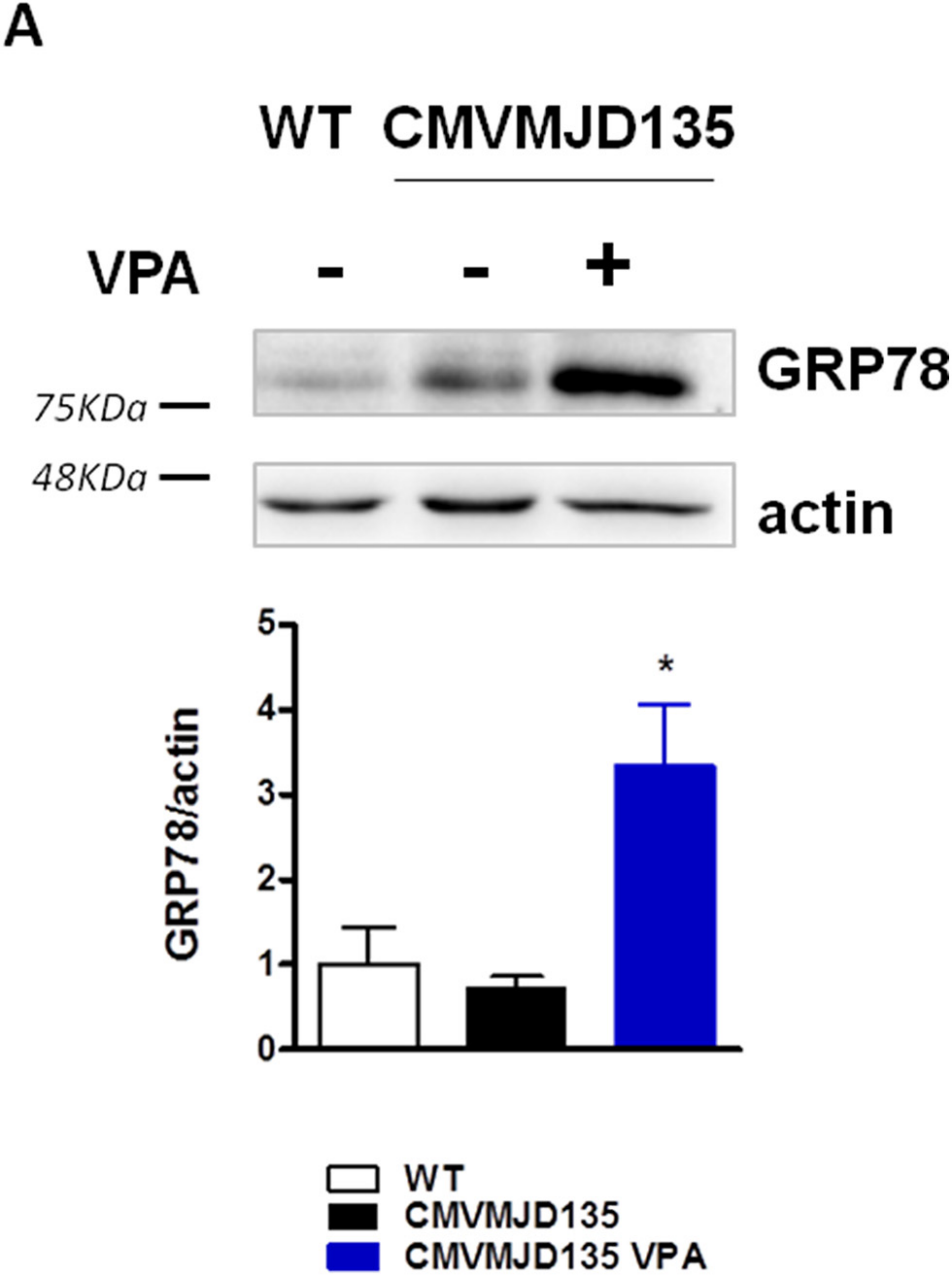
Cerebellum western-blot and quantification of GRP78 protein levels in 30 week-old WT, VPA-treated and non-treated CMVMJD135 mice. GRP78 protein induction in CMVMJD135 animals upon VPA treatment. Bars represent the mean ± SEM (n = 4 for each group), * represent p<0.05, One-way ANOVA.

One of the neuroprotective actions described for VPA is its ability to increase GRP78 protein levels, through HDAC inhibition [15]. GRP78, also known as binding immunoglobulin protein (BiP), is a stress chaperone protein found in the lumen of the endoplasmic reticulum (ER) which binds newly synthesized proteins as they are translocated into the ER, and keeps them in a competent state for subsequent folding and oligomerization [27]. We specifically investigated the potential role of HDAC inhibition by monitoring the GRP78 protein levels induction upon VPA treatment. We observe a striking increase in GRP78 protein levels in CMVMJD135-treated animals when compared to non-treated animals, in the cerebellum at 30 weeks of age (Fig 6).

## Discussion

In the past years, the use of VPA as a treatment for neurodegenerative disease models has been shown to improve neurological phenotypes, decrease cell degeneration and toxicity, together with the increase of histone acetylation and subsequent gene transcription activation [28–33]. The dosage of VPA of 200mg/kg used in this pre-clinical trial was previously described in a pre-clinical trial ALS, in which neuroprotective and histone acetylation effects were shown [34,35]. Here, we also show that five days treatment with 200mg/kg of VPA was able to exert a trend towards an increase in histone acetylation in cerebellum and a tendency to increase GADD45α and BIM mRNA levels, which may be used as VPA target engagement [36–38]. The acute treatment and/or high variability between animals may account for the lack of statistical differences between groups. Acute VPA treated animals presented therapeutic levels of 252 ± 15.8 μmol/L and 25.7 ± 8.7 μmol/L in plasma, after 30 and 120 minutes post-injection, respectively, which was within the usually accepted therapeutic range of VPA. Therefore, we performed a pre-clinical trial with five consecutive day treatment starting at 5 and ending at 30 weeks of age in CMVMJD135 mouse model. Animals in the pre-clinical trial were always maintained in an appropriate and healthy environment avoiding the development of any stressful condition that may interfere with their motor performance. Although previous findings by several groups suggested a therapeutic effect for VPA in cellular and invertebrate animal models of SCA3, in the present study, chronic VPA treatment of CMVMJD135 mice lead to a late and limited improvement in the motor performance, given by the beam balance, motor swimming, rotarod and spontaneous locomotor activity tests. For other general health and neuromuscular function, VPA-treatment had only marginal or even no effects comparing to vehicle-treated transgenic animals. In addition, the dosage used in this study is not considered toxic for mice and is already described as safe in the literature [18,34,35,39]. Nevertheless, chronic VPA treatment at similar dosage was already described to reduce the body mass gain in WT animals [34], even though chronically treated human patients are known to increase their body weight upon VPA treatment [40–42]. Metabolic differences and biological system diversity could be the reason for these contradictory observations.

At the pathological level, we examined the presence of ATXN3 neuronal nuclear inclusions in facial nuclei (7N) and lateral reticular nuclei (LRt), two regions described to be affected in MJD human patients [43,44], as well as in CMVMJD135 mouse model [22]. No differences were observed in the amount of nuclear neuronal inclusions of ATXN3 in both VPA-treated and non-treated CMVMJD135 mice.

Additionally, astrogliosis is a consistent pathological phenotype of CMVMJD135 mouse model and human patients, which was also not mitigated by VPA chronic treatment.

The overexpression of specific chaperones has been shown to allow protection against cellular damage and/or death caused from an extensive group of agents and conditions including cytotoxic chemicals [45], oxidative stress [46] and ER stress [27]. Here we show that the induction of GRP78 protein levels upon VPA chronic treatment, whose overexpression may be neuroprotective in proteinopathies, including MJD [47,48], could be one of its cytoprotective actions in the MJD context, enhancing the folding capacity of the ER. The induction of GRP78 also indicates VPA target engagement during the chronic treatment in the pre-clinical trial, as this protein is known to be induced by VPA [15]. Although we did not observe a statistical difference in the CMVMJD135 animals when comparing to WT, enhancing the expression of this molecular chaperone in the cerebellum of these mice could be one of the neuroprotective mechanisms responsible for the late and mild improvement of the CMVMJD135 animal motor performance.

The observed beneficial effects of VPA were transient, occurred mostly later in life and thus at an advanced stage of disease (between 22 and 30 weeks of age), mainly in behavioral tests more related with motor coordination. These results are comparable to some extent with previous findings, in which we have shown a significant reduction in neurological dysfunction in a *C*. *elegans* model of MJD after VPA treatment that was more relevant later in the worm’s life (day10) in spite of early treatment [21]. Previous evidence also suggested a protective role for VPA in the context of MJD, both in cell and *Drosophila* models, by attenuating mutant ATXN3 induced cell toxicity and alleviating polyQ-induced phenotypic abnormalities, without major impact on ATXN3 inclusion [19,20]; in *C*. *elegans*, there was some improvement of aggregation, but less prominent than that observed for other compounds, for instance Hsp90 inhibitors [21].

Although the effects observed in our mouse model were not striking, only one dosage of VPA was tested; thus, the possibility of testing other dosages, far from toxic and lethal ones [49,50], should be considered, as they could exert more pronounced effects. Moreover, the complex activity and a broad range of VPA effects also create the need for further clarification of the effects of this drug not only at the symptom level, but also molecular and pathological levels in the CMVMJD135 mouse model. In fact, and although the use of HDACi’s in the context of polyQ diseases has showed promising results, the evidence for a globally decreased histone acetylation is not fully consistent [51], and this strategy still lacks some target specificity/selectivity [52] and requires a more in depth study of the mechanisms of action of these compounds in the central nervous system. Furthermore, chronic VPA treatment in human patients can produce some side effects, such as weight gain [53,54], decreased reproductive potential [55,56] and increased susceptibility to birth defects [57–59]. Nevertheless, the strategy of re-purposing FDA/EMA-approved molecules, as VPA, could be of benefit for MJD and other rare diseases lacking effective therapies. Additionally, there is still an open window for different VPA dosages to be tested and since in the past years, a growing number of efforts are being developed for the formulation of a new generation of more selective and specific compounds this could be useful for the treatment not only of MJD, but also of other polyglutamine diseases.

## Material and Methods

### Ethics statement

All animal procedures were conducted in accordance with European regulations (European Union Directive 86/609/EEC). Animal facilities and the people directly involved in animal experiments (SE, SDS, ANC and ASF) were certified by the Portuguese regulatory entity—Direcção Geral de Alimentação e Veterinária. All of the protocols performed were approved by the Ethics Subcommittee for Life and Health Sciences of the Life and Health Sciences Research Institute, University of Minho. All experiments were designed with commitment to the principles of refinement, reduction, and replacement and performed according to the FELASA guidelines to minimize discomfort, stress, and pain to the animals, with defined humane endpoints [60]. Humane endpoints for the preclinical trial were defined as 20% reduction of the body weight, inability to reach food and water, presence of wounds in the body and dehydration), however they were not needed as the study period was conceived to include ages at which animals do not reach these endpoints.

### Transgenic mice model and drug administration

We used the CMVMJD135 (background C57BL/6) mouse model, expressing an expanded version of the human MJD1-1 cDNA (the 3 UIMs-containing variant of ATXN3) under the regulation of the CMV promoter, ubiquitously and at near-endogenous levels [22]. These animals show a slowly progressive motor phenotype and CNS pathology consistent with that of MJD patients [22]. Male transgenic and non-transgenic drug- and placebo- treated animals were sequentially assigned and housed at weaning in groups of 5 animals in filter-topped polysulfone cages 267 × 207 × 140 mm (370 cm^2^ floor area) (Tecniplast, Buguggiate, Italy), with corncob bedding (Scobis Due, Mucedola SRL, Settimo Milanese, Italy) in a conventional animal facility. DNA extraction, animal genotyping and CAG repeat size analyses were performed as previously described [61], with the mean repeat size (±SD) for all mice of (133±1). Male littermates wild-type (WT) animals were used as controls. All animals were maintained under standard laboratory conditions: an artificial 12 h light/dark cycle (lights on from 8:00 to 20:00 h), with an ambient temperature of 21±1°C and a relative humidity of 50–60%; the mice were given a standard diet (4RF25 during the gestation and postnatal periods, and 4RF21 after weaning, Mucedola SRL, Settimo Milanese, Italy) and water *ad libitum*. We administered Valproic acid sodium salt (PG-4543, Sigma) during five consecutive days per week through intraperitoneal injection (i.p), in a dosage of 200mg/kg dissolved in 0,9% saline. Control animals were given a placebo of injection buffer (0.9% NaCl) with the same frequency. Treatment was initiated at five weeks of age, i.e. one week before the onset of the first neurological symptoms, until 30 weeks of age of the pre-clinical trial. For a pilot study, WT animals were treated for 5 consecutive days with i.p. injections of VPA at 200mg/kg or saline.

### Western-blot analysis

Cerebellum tissues were thawed and homogenized with a Potter-Elvejhem 377 homogenizer with a Teflon pestle, at 300 rpm, in lysis buffer (150 mM NaCl, 50 mM Tris, 5 mM EGTA, 1% Triton X-100, 0.5% sodium deoxycholate, 0.1% SDS, pH 7.5) supplemented with 100 nM okadaic acid, 25 mM NaF, 1 mM Na3VO4, 1 mM DTT, 1 mM PMSF, 1 μg/mL of protease inhibitor cocktail (chymostatin, pepstatin A, leupeptin and antipain), 1 μM trichostatin A (HDACs inhibitor) and 10 mM nicotinamide (sirtuins inhibitor). The homogenates were then sonicated for 15 s and centrifuged at 20,800 g for 10 min to remove cell debris. The pellet was discarded, the supernatant (total extract) was collected and protein content quantified by Bio-Rad protein assay (Bio-Rad). Total extracts were denatured with denaturing buffer (50 mM Tris-HCl pH 6.8, 2% SDS, 5% glycerol, 600 mM DTT, 0.01% bromophenol blue) at 95°C, for 5 min. Equivalent amounts of protein (30μg) were separated on a 15% SDS-PAGE gel electrophoresis and electroblotted onto polyvinylidene difuoride (PVDF) membranes. The membranes were blocked for 1 h in Tris-buffered saline (TBS) solution containing 0.1% Tween (TBS-Tween) and 5% BSA, followed by an overnight incubation with primary antibodies (rabbit anti-acH3 (1:1000, Milipore), rabbit anti-Lamin B1 (1:1000, Abcam) rabbit anti-GRP78 (1:1000, Abcam) and mouse anti-actin (1:5000, Ambion), at 4°C, with gentle agitation. Membranes were then washed 3 times, for 10 min, with TBS-Tween, and incubated with secondary antibodies conjugated with alkaline phosphatase (1:10000), for 1 h, at room temperature, with gentle agitation. Immunoreactive bands were visualized by alkaline phosphatase activity after incubation with ECL substrate, in a ChemiDoc Imaging System (Bio-Rad). Bands were quantified using the Image Lab software (Bio-Rad).

### Gene expression quantification (qRT-PCR)

Cerebellum total RNA was isolated from 18 week-old CMVMJD135 littermate mice, vehicle- and VPA-treated (n = 4 for each group) using TRIZOL (15596–026, Invitrogen, Calrsbad, USA) according to the manufacturer’s protocol. RNA samples were treated with DNase I (EN0525, Thermo Scientific®, USA) according to the manufacturer’s protocol. First-strand cDNA, synthesized with iScript cDNA Synthesis kit (#170–8891, Bio-Rad, USA) was amplified by quantitative reverse-transcriptase PCR (qRT-PCR) as previously described [22]. The following primers were used for expression quantification: GADD45α (F 5-AGACCGAAAGGATGGACACG-3’); GADD45α (R 5’-TGACTCCGAGCCTTGCTGA-3’); BIM (F 5’-CGGATCGGAGACGAGTTCA-3’); BIM (R 5’-TTCAGCCTCGCGGTAATCA-3’); B2m (F 5’-CCTTCAGCAAGGACTGGTCT-3’ and B2m (R 5’-TCTCGATCCCAGTAGACGGT-3’). Primers were designed using PRIMER-BLAST (http://www.ncbi.nlm.nih.gov/tools/primer-blast/).

### Behavioral analysis

Behavioral analysis was performed during the diurnal period in groups of 5 male animals per cage including CMVMJD135 hemizygous transgenic mice and WT littermates (n = 10–15 per genotype) treated and non-treated with VPA. All behavioral tests started in a pre-symptomatic stage of the disease (4 weeks of age) and were conducted until an age at which the phenotype is fully established (30 weeks) [22]. The animals were weighed one week before the start of drug treatment (4 weeks) and then every two weeks until 30 weeks of age (Fig 2).

#### Neurological examination (based on SHIRPA protocol)

Based on the SHIRPA protocol we established an adapted protocol for phenotypic assessment applied since 4 weeks until 24 weeks of age, in which we used the tests for which, based on our previous experience, CMVMJD135 mice usually present significant phenotypic alterations [22]. We assessed motor function through the spontaneous activity test, by counting wall-leanings during five minutes, and locomotor activity in which we counted the number of squares travelled over 30 secs, in an arena (55×33×18 cm) with 15 labeled squares. Other observational measurements included tremors and limb clasping, in which we suspended the animal by the tail and classified the extensor reflexes. In this protocol we also included the hanging wire test, as a measure of muscle strength and fine motor coordination of the paws. This protocol was adjusted in order to minimize animal handling and to generate uniformity in waiting times between the tests [62].

#### Footprint analysis

To evaluate the dragging of the paws, the footprint test was used since 10 weeks of age. To obtain footprints, the hind‐ and forepaws of the mice were coated with black and red non‐toxic paints, respectively. We used a clean rectangular paper sheet placed on the floor of the runway for each run. The animals were allowed to walk along a 100‐cm‐long × 4.2 cm width × 10 cm height corridor in the direction of an enclosed black box. Each animal was allowed to achieve one valid trial per age. To evaluate the severity of foot-dragging through age the footprinting pattern was classified at each time point considering six consecutive steps (0 = absent dragging, up to three steps; 1 = moderate dragging, less than three steps out of six; 2 = severe dragging, all steps out of six show dragging). The stride length was also measured through the footprinting pattern by measuring the length between three consecutive steps.

#### Motor swimming test

To assess swimming movement coordination, the time that animals take to reach a safe platform at the end of a container (60 cm distance) with 15 cm depth of water at 24‐26°C was recorded bi-weekly since 22 weeks of age. The protocol consisted of 2 days of training with three trial followed by three days of test with two trials as previously described [22,63].

#### Beam walk test

Balance and fine motor coordination of mice were assessed by measuring the ability of the mice to traverse, without falling, a graded series of narrow beams to reach an enclosed safety platform as previously described [22,63]. During training, mice were placed at the start of the 12 mm square beam and trained over 3 days (3 trials per day) to traverse the beam to the safe platform. On the fourth day, they were tested in the training beam (12 mm square) and 11 mm round beam (2 trials per beam).

#### Rotarod test

To evaluate motor skill learning and coordination with another paradigm, mice were tested in a rotarod apparatus (TSE systems, Bad Homburg, Germany). The protocol is comprised of 3 training days at a constant speed (15 rpm) for a maximum of 60 s in four trials, with a 10 min interval between each trial. On the fourth day, animals were tested for each of 6 different speeds (5 rpm, 8 rpm, 15 rpm, 20 rpm, 24 rpm and 31 rpm) for a maximum of 60s in two trials, with a 10-min-long interval between each trial, as previously described [61].

### Immunohistochemistry and quantification of ataxin-3 neuronal inclusions and astrogliosis

Thirty week-old WT and CMVMJD135 littermate mice, VPA-treated and non-treated (n = 4 for each group) were deeply anesthetized- with a mixture of ketamine hydrochloride (150 mg/kg) plus medetomidine (0.3 mg/kg) and transcardially perfused with phosphate-buffered saline (PBS) followed by 4% paraformaldehyde (PFA) (Panreac, USA). Brains were removed and post fixed overnight in PFA 4% and embedded in paraffin. Slides with 4-μm-thick paraffin sections were subjected to antigen retrieval (Buffer Citrate, 1M) and then incubated with mouse anti-ATXN3 (1H9) (1:1000, MAB5360, Milipore) and GFAP (1:500, Z0334, Dako corporation) which were detected by incubation with a biotinylated anti-polyvalent antibody, followed by detection through biotin-streptavidin coupled to horseradish peroxidase and reaction with the DAB (3, 3'-diaminobenzidine) substrate (Lab VisionTM Ultra-VisionTM Detection kit, Thermo Scientific). The slides were counterstained with 25% hematoxylin according to standard procedures. ATXN3 positive inclusions in the facial motor nucleus (7N) and lateral reticular nucleus (LRt), and GFAP positive cells in substantia nigra (SN) of vehicle or VPA-treated animals (n = 4 for each conditions, 4 slides per animal) were quantified and normalized for total area using the Olympus BX51 stereological microscope (Olympus, Japan) and the Visiopharma integrator system software (Visopharm, Denmark) as previously described [22]. The total area of 7N,LRT and SN were chosen based on the mouse brain atlas [64].

### Determination of Valproic acid Plasma Levels

The plasma valproic acid levels were measured applying the VALP assay using the Dimension Vista® System (VALP Flex® reagent cartridge)–SIEMENS.

### Statistical analysis

The experimental unit used in this study was a single animal. Experimental design was based on power analyses for optimization of sample size [65]. Mouse sample size calculations were performed for each behavioral test and pathological analyses assuming a power of 0.8 and a significance level of p < 0.05. The effect size was calculated aiming at detecting 50% improvement. We used n = 10 to 15 per genotype/treatment for behavioral tests, and a group size of four animals per group for quantification of ATXN3 NIIs analysis. Data was analyzed through the non-parametric Mann-Whitney U-test when variables were non-continuous or when a continuous variable did not present a normal distribution (Kolmogorov-Smirnov test, p<0.05) (Rotarod). Continuous variables with normal distributions and with homogeneity of variance (Levene’s test) were analyzed with Repeated-Measures ANOVA for longitudinal multiple comparisons, using Tukey test for post-hoc comparisons and One-way ANOVA for paired comparisons Non-continuous categorical variables were analyzed through Chi-Square Fisher exact test. All statistical analyses were performed using SPSS 22.0 (SPSS Inc., Chicago, IL) and G-Power 3.1.9.2 (University Kiel, Germany). A critical value for significance of *P* < 0.05 was used throughout the study. Values were expressed as mean ± SEM for continuous variables and as percentages for non‐continuous variables.

## Supporting Information

S1 Checklist NC3Rs ARRIVE Guidelines Checklist is provided as supporting information. (DOCX)

S1 Fig Balance and motor coordination were improved at later stages upon VPA treatment given by the balance beam and motor swimming performance. (A) Amelioration of balance and motor coordination at 24 weeks of age in 11 mm circle and (B) 12 mm square beams in beam walk test; (C) Motor swimming coordination improvement; (D,E) no improvement in increasing rotations in Rotarod were observed between VPA-treated and non-treated CMVMJD135. Bars represent the mean ± SEM (WT veh, n = 10; WT VPA, n = 15, CMVMJD135 vehicle, n = 10, CMVMJD135 VPA, n = 13), * represent p<0.05, ** represent p<0.01 and *** represent p<0.001, black asterisks represents the difference between WT and CMVMJD135, blue asterisks represents the difference between non-treated and VPA-treated CMVMJD135, (Repeated-measures ANOVA, Tukey correction for continuous variables, One-Way ANOVA for differences between groups in specific ages of the continuous variables and Mann-Whitney U test for continuous variables without normal distribution (Rotarod)).
(TIF)

## References

[1] Bauer PO, Nukina N. The pathogenic mechanisms of polyglutamine diseases and current therapeutic strategies. J Neurochem. 2009;110: 1737–1765. 10.1111/j.1471-4159.2009.06302.x 19650870

[2] Ross CA, Margolis RL, Becher MW, Wood JD, Engelender S, Cooper JK, et al Pathogenesis of neurodegenerative diseases associated with expanded glutamine repeats: new answers, new questions. Prog Brain Res. 1998;117: 397–419. 9932422 10.1016/s0079-6123(08)64029-7

[3] Kawaguchi Y, Okamoto T, Taniwaki M, Aizawa M, Inoue M, Katayama S, et al CAG expansions in a novel gene for Machado-Joseph disease at chromosome 14q32.1. Nat Genet. 1994;8: 221–228. 10.1038/ng1194-221 7874163

[4] Rüb U, Brunt ER, Deller T. New insights into the pathoanatomy of spinocerebellar ataxia type 3 (Machado-Joseph disease). Curr Opin Neurol. 2008;21: 111–116. 10.1097/WCO.0b013e3282f7673d 18317266

[5] Barbeau A, Roy M, Cunha L, De Vincente AN, Rosenberg RN, Nyhan WL, et al The natural history of Machado-Joseph disease. An analysis of 138 personally examined cases. Can J Neurol Sci. 1984;11: 510–525. 6509398 10.1017/s0317167100034983

[6] Coutinho P, Andrade C. Autosomal dominant system degeneration in Portuguese families of the Azores Islands. A new genetic disorder involving cerebellar, pyramidal, extrapyramidal and spinal cord motor functions. Neurology. 1978;28: 703–709. 566869 10.1212/wnl.28.7.703

[7] Lima L, Coutinho P. Clinical criteria for diagnosis of Machado-Joseph disease: report of a non-Azorena Portuguese family. Neurology. 1980;30: 319–322. 7189034 10.1212/wnl.30.3.319

[8] Hoffner G, Djian P. Polyglutamine Aggregation in Huntington Disease: Does Structure Determine Toxicity? Mol Neurobiol. 2014; 10.1007/s12035-014-8932-1 25336039

[9] Seidel K, Den Dunnen WFA, Schultz C, Paulson H, Frank S, De Vos RA, et al Axonal inclusions in spinocerebellar ataxia type 3. Acta Neuropathol. 2010;120: 449–460. 10.1007/s00401-010-0717-7 20635090 PMC2923324

[10] Evert BO, Schelhaas J, Fleischer H, De Vos RAI, Brunt ER, Stenzel W, et al Neuronal intranuclear inclusions, dysregulation of cytokine expression and cell death in spinocerebellar ataxia type 3. Clin Neuropathol. 2006;25: 272–281. 17140157

[11] Rüb U, De Vos RAI, Brunt ER, Sebestény T, Schöls L, Auburger G, et al Spinocerebellar ataxia type 3 (SCA3): thalamic neurodegeneration occurs independently from thalamic ataxin-3 immunopositive neuronal intranuclear inclusions. Brain Pathol. 2006;16: 218–227. 10.1111/j.1750-3639.2006.00022.x 16911479 PMC8095748

[12] Weber JJ, Sowa AS, Binder T, Hübener J. From Pathways to Targets: Understanding the Mechanisms behind Polyglutamine Disease. BioMed Research International. 2014;2014: 1–22. 10.1155/2014/701758 PMC4189765 25309920

[13] Rosenberg G. The mechanisms of action of valproate in neuropsychiatric disorders: can we see the forest for the trees? Cell Mol Life Sci. 2007;64: 2090–2103. 10.1007/s00018-007-7079-x 17514356 PMC11149473

[14] Perucca E. Pharmacological and therapeutic properties of valproate: a summary after 35 years of clinical experience. CNS Drugs. 2002;16: 695–714. 12269862 10.2165/00023210-200216100-00004

[15] Shi Y, Gerritsma D, Bowes AJ, Capretta A, Werstuck GH. Induction of GRP78 by valproic acid is dependent upon histone deacetylase inhibition. Bioorg Med Chem Lett. 2007;17: 4491–4494. 10.1016/j.bmcl.2007.06.006 17566732

[16] Zádori D, Geisz A, Vámos E, Vécsei L, Klivényi P. Valproate ameliorates the survival and the motor performance in a transgenic mouse model of Huntington’s disease. Pharmacol Biochem Behav. 2009;94: 148–153. 10.1016/j.pbb.2009.08.001 19698736

[17] Tsai L-K, Tsai M-S, Ting C-H, Li H. Multiple therapeutic effects of valproic acid in spinal muscular atrophy model mice. J Mol Med. 2008;86: 1243–1254. 10.1007/s00109-008-0388-1 18649067

[18] Feng H-L, Leng Y, Ma C-H, Zhang J, Ren M, Chuang D-M. Combined lithium and valproate treatment delays disease onset, reduces neurological deficits and prolongs survival in an amyotrophic lateral sclerosis mouse model. Neuroscience. 2008;155: 567–572. 10.1016/j.neuroscience.2008.06.040 18640245 PMC2709275

[19] Yi J, Zhang L, Tang B, Han W, Zhou Y, Chen Z, et al Sodium valproate alleviates neurodegeneration in SCA3/MJD via suppressing apoptosis and rescuing the hypoacetylation levels of histone H3 and H4. PLoS ONE. 2013;8: e54792 10.1371/journal.pone.0054792 23382971 PMC3557284

[20] Lin XP, Feng L, Xie CG, Chen DB, Pei Z, Liang XL, et al Valproic acid attenuates the suppression of acetyl histone H3 and CREB activity in an inducible cell model of Machado-Joseph disease. Int J Dev Neurosci. 2014;38: 17–22. 10.1016/j.ijdevneu.2014.07.004 25068645

[21] Teixeira-Castro A, Ailion M, Jalles A, Brignull HR, Vilaça JL, Dias N, et al Neuron-specific proteotoxicity of mutant ataxin-3 in C. elegans: rescue by the DAF-16 and HSF-1 pathways. Hum Mol Genet. 2011;20: 2996–3009. 10.1093/hmg/ddr203 21546381 PMC3131043

[22] Silva-Fernandes A, Duarte-Silva S, Neves-Carvalho A, Amorim M, Soares-Cunha C, Oliveira P, et al Chronic treatment with 17-DMAG improves balance and coordination in a new mouse model of Machado-Joseph disease. Neurotherapeutics. 2014;11: 433–449. 10.1007/s13311-013-0255-9 24477711 PMC3996110

[23] Sultan FA, Sweatt JD. The role of the Gadd45 family in the nervous system: a focus on neurodevelopment, neuronal injury, and cognitive neuroepigenetics. Adv Exp Med Biol. 2013;793: 81–119. 10.1007/978-1-4614-8289-5_6 24104475

[24] Akhtar RS, Ness JM, Roth KA. Bcl-2 family regulation of neuronal development and neurodegeneration. Biochimica et Biophysica Acta (BBA)—Molecular Cell Research. 2004;1644: 189–203. 10.1016/j.bbamcr.2003.10.013 14996503

[25] Yamauchi J, Miyamoto Y, Murabe M, Fujiwara Y, Sanbe A, Fujita Y, et al Gadd45a, the gene induced by the mood stabilizer valproic acid, regulates neurite outgrowth through JNK and the substrate paxillin in N1E-115 neuroblastoma cells. Exp Cell Res. 2007;313: 1886–1896. 10.1016/j.yexcr.2007.02.019 17428471

[26] Xie C, Edwards H, Xu X, Zhou H, Buck SA, Stout ML, et al Mechanisms of synergistic antileukemic interactions between valproic acid and cytarabine in pediatric acute myeloid leukemia. Clin Cancer Res. 2010;16: 5499–5510. 10.1158/1078-0432.CCR-10-1707 20889917 PMC3018695

[27] Morris JA, Dorner AJ, Edwards CA, Hendershot LM, Kaufman RJ. Immunoglobulin binding protein (BiP) function is required to protect cells from endoplasmic reticulum stress but is not required for the secretion of selective proteins. J Biol Chem. 1997;272: 4327–4334. 9020152 10.1074/jbc.272.7.4327

[28] Steffan JS, Bodai L, Pallos J, Poelman M, McCampbell A, Apostol BL, et al Histone deacetylase inhibitors arrest polyglutamine-dependent neurodegeneration in Drosophila. Nature. 2001;413: 739–743. 10.1038/35099568 11607033

[29] Ferrante RJ, Kubilus JK, Lee J, Ryu H, Beesen A, Zucker B, et al Histone deacetylase inhibition by sodium butyrate chemotherapy ameliorates the neurodegenerative phenotype in Huntington’s disease mice. J Neurosci. 2003;23: 9418–9427. 14561870 10.1523/JNEUROSCI.23-28-09418.2003 PMC6740577

[30] Hockly E, Richon VM, Woodman B, Smith DL, Zhou X, Rosa E, et al Suberoylanilide hydroxamic acid, a histone deacetylase inhibitor, ameliorates motor deficits in a mouse model of Huntington’s disease. Proc Natl Acad Sci USA. 2003;100: 2041–2046. 10.1073/pnas.0437870100 12576549 PMC149955

[31] Chou A-H, Chen S-Y, Yeh T-H, Weng Y-H, Wang H-L. HDAC inhibitor sodium butyrate reverses transcriptional downregulation and ameliorates ataxic symptoms in a transgenic mouse model of SCA3. Neurobiol Dis. 2011;41: 481–488. 10.1016/j.nbd.2010.10.019 21047555

[32] Hahnen E, Hauke J, Tränkle C, Eyüpoglu IY, Wirth B, Blümcke I. Histone deacetylase inhibitors: possible implications for neurodegenerative disorders. Expert Opin Investig Drugs. 2008;17: 169–184. 10.1517/13543784.17.2.169 18230051

[33] Minamiyama M, Katsuno M, Adachi H, Waza M, Sang C, Kobayashi Y, et al Sodium butyrate ameliorates phenotypic expression in a transgenic mouse model of spinal and bulbar muscular atrophy. Hum Mol Genet. 2004;13: 1183–1192. 10.1093/hmg/ddh131 15102712

[34] Rouaux C, Panteleeva I, René F, Gonzalez de Aguilar J-L, Echaniz-Laguna A, Dupuis L, et al Sodium valproate exerts neuroprotective effects in vivo through CREB-binding protein-dependent mechanisms but does not improve survival in an amyotrophic lateral sclerosis mouse model. J Neurosci. 2007;27: 5535–5545. 10.1523/JNEUROSCI.1139-07.2007 17522299 PMC6672753

[35] Hoffmann K, Czapp M, Löscher W. Increase in antiepileptic efficacy during prolonged treatment with valproic acid: role of inhibition of histone deacetylases? Epilepsy Res. 2008;81: 107–113. 10.1016/j.eplepsyres.2008.04.019 18538545

[36] Yamauchi J, Miyamoto Y, Murabe M, Fujiwara Y, Sanbe A, Fujita Y, et al Gadd45a, the gene induced by the mood stabilizer valproic acid, regulates neurite outgrowth through JNK and the substrate paxillin in N1E-115 neuroblastoma cells. Exp Cell Res. 2007;313: 1886–1896. 10.1016/j.yexcr.2007.02.019 17428471

[37] Stauber RH, Knauer SK, Habtemichael N, Bier C, Unruhe B, Weisheit S, et al A combination of a ribonucleotide reductase inhibitor and histone deacetylase inhibitors downregulates EGFR and triggers BIM-dependent apoptosis in head and neck cancer. Oncotarget. 2012;3: 31–43. 22289787 10.18632/oncotarget.430 PMC3292890

[38] Mologni L, Cleris L, Magistroni V, Piazza R, Boschelli F, Formelli F, et al Valproic acid enhances bosutinib cytotoxicity in colon cancer cells. Int J Cancer. 2009;124: 1990–1996. 10.1002/ijc.24158 19123474

[39] Tremolizzo L, Carboni G, Ruzicka WB, Mitchell CP, Sugaya I, Tueting P, et al An epigenetic mouse model for molecular and behavioral neuropathologies related to schizophrenia vulnerability. Proc Natl Acad Sci USA. 2002;99: 17095–17100. 10.1073/pnas.262658999 12481028 PMC139275

[40] Corman CL, Leung NM, Guberman AH. Weight gain in epileptic patients during treatment with valproic acid: a retrospective study. Can J Neurol Sci. 1997;24: 240–244. 9276111 10.1017/s0317167100021879

[41] Martin CK, Han H, Anton SD, Greenway FL, Smith SR. Effect of valproic acid on body weight, food intake, physical activity and hormones: results of a randomized controlled trial. J Psychopharmacol (Oxford). 2009;23: 814–825. 10.1177/0269881108091595 18583434 PMC2753432

[42] Verrotti A, D’Egidio C, Mohn A, Coppola G, Chiarelli F. Weight gain following treatment with valproic acid: pathogenetic mechanisms and clinical implications. Obes Rev. 2011;12: e32–43. 10.1111/j.1467-789X.2010.00800.x 20880119

[43] Rüb U, Brunt ER, Petrasch-Parwez E, Schöls L, Theegarten D, Auburger G, et al Degeneration of ingestion-related brainstem nuclei in spinocerebellar ataxia type 2, 3, 6 and 7. Neuropathol Appl Neurobiol. 2006;32: 635–649. 10.1111/j.1365-2990.2006.00772.x 17083478

[44] Rüb U, De Vos RAI, Schultz C, Brunt ER, Paulson H, Braak H. Spinocerebellar ataxia type 3 (Machado-Joseph disease): severe destruction of the lateral reticular nucleus. Brain. 2002;125: 2115–2124. 12183356 10.1093/brain/awf208

[45] Reddy RK, Mao C, Baumeister P, Austin RC, Kaufman RJ, Lee AS. Endoplasmic reticulum chaperone protein GRP78 protects cells from apoptosis induced by topoisomerase inhibitors: role of ATP binding site in suppression of caspase-7 activation. J Biol Chem. 2003;278: 20915–20924. 10.1074/jbc.M212328200 12665508

[46] Liu H, Bowes RC, Van de Water B, Sillence C, Nagelkerke JF, Stevens JL. Endoplasmic reticulum chaperones GRP78 and calreticulin prevent oxidative stress, Ca2+ disturbances, and cell death in renal epithelial cells. J Biol Chem. 1997;272: 21751–21759. 9268304 10.1074/jbc.272.35.21751

[47] Gorbatyuk MS, Gorbatyuk OS. The Molecular Chaperone GRP78/BiP as a Therapeutic Target for Neurodegenerative Disorders: A Mini Review. J Genet Syndr Gene Ther. 2013;4 10.4172/2157-7412.1000128 23750325 PMC3674964

[48] Pereira CMF. Crosstalk between Endoplasmic Reticulum Stress and Protein Misfolding in Neurodegenerative Diseases. ISRN Cell Biology. 2013;2013: 1–22. 10.1155/2013/256404

[49] Goodwin DG, Strobl J, Mitchell SM, Zajac AM, Lindsay DS. Evaluation of the mood-stabilizing agent valproic acid as a preventative for toxoplasmosis in mice and activity against tissue cysts in mice. J Parasitol. 2008;94: 555–557. 10.1645/GE-1331.1 18564764

[50] Löscher W. Pharmacological, toxicological and neurochemical effects of delta 2(E)-valproate in animals. Pharm Weekbl Sci. 1992;14: 139–143. 1502015 10.1007/BF01962705

[51] Valor LM, Guiretti D, Lopez-Atalaya JP, Barco A. Genomic landscape of transcriptional and epigenetic dysregulation in early onset polyglutamine disease. J Neurosci. 2013;33: 10471–10482. 10.1523/JNEUROSCI.0670-13.2013 23785159 PMC6618595

[52] Kazantsev AG, Thompson LM. Therapeutic application of histone deacetylase inhibitors for central nervous system disorders. Nat Rev Drug Discov. 2008;7: 854–868. 10.1038/nrd2681 18827828

[53] Wirrell EC. Valproic acid-associated weight gain in older children and teens with epilepsy. Pediatr Neurol. 2003;28: 126–129. 12699863 10.1016/s0887-8994(02)00505-2

[54] Grosso S, Mostardini R, Piccini B, Balestri P. Body mass index and serum lipid changes during treatment with valproic acid in children with epilepsy. Ann Pharmacother. 2009;43: 45–50. 10.1345/aph.1L414 19066323

[55] Isojärvi J. Disorders of reproduction in patients with epilepsy: antiepileptic drug related mechanisms. Seizure. 2008;17: 111–119. 10.1016/j.seizure.2007.11.007 18164216

[56] Verrotti A, Loiacono G, Laus M, Coppola G, Chiarelli F, Tiboni GM. Hormonal and reproductive disturbances in epileptic male patients: emerging issues. Reprod Toxicol. 2011;31: 519–527. 10.1016/j.reprotox.2011.02.002 21338669

[57] Clayton-Smith J, Donnai D. Fetal valproate syndrome. J Med Genet. 1995;32: 724–727. 8544193 10.1136/jmg.32.9.724 PMC1051674

[58] Genton P, Semah F, Trinka E. Valproic acid in epilepsy: pregnancy-related issues. Drug Saf. 2006;29: 1–21. 16454531 10.2165/00002018-200629010-00001

[59] Ornoy A. Valproic acid in pregnancy: how much are we endangering the embryo and fetus? Reprod Toxicol. 2009;28: 1–10. 10.1016/j.reprotox.2009.02.014 19490988

[60] FELASA working group on revision of guidelines for health monitoring of rodents and rabbits, Mähler Convenor M, Berard M, Feinstein R, Gallagher A, Illgen-Wilcke B, et al FELASA recommendations for the health monitoring of mouse, rat, hamster, guinea pig and rabbit colonies in breeding and experimental units. Lab Anim. 2014;48: 178–192. 10.1177/0023677213516312 24496575

[61] Silva-Fernandes A, Costa M do C, Duarte-Silva S, Oliveira P, Botelho CM, Martins L, et al Motor uncoordination and neuropathology in a transgenic mouse model of Machado-Joseph disease lacking intranuclear inclusions and ataxin-3 cleavage products. Neurobiol Dis. 2010;40: 163–176. 10.1016/j.nbd.2010.05.021 20510362

[62] Rafael JA, Nitta Y, Peters J, Davies KE. Testing of SHIRPA, a mouse phenotypic assessment protocol, on Dmd(mdx) and Dmd(mdx3cv) dystrophin-deficient mice. Mamm Genome. 2000;11: 725–728. 10967129 10.1007/s003350010149

[63] Carter RJ, Lione LA, Humby T, Mangiarini L, Mahal A, Bates GP, et al Characterization of progressive motor deficits in mice transgenic for the human Huntington’s disease mutation. J Neurosci. 1999;19: 3248–3257. 10191337 10.1523/JNEUROSCI.19-08-03248.1999 PMC6782264

[64] Franklin KBJ. Paxinos and Franklin’s The mouse brain in stereotaxic coordinates Fourth edition Amsterdam: Academic Press, an imprint of Elsevier; 2013.

[65] Zar JH. Biostatistical analysis 4th ed. Upper Saddle River, N.J: Prentice Hall; 1999.

