# Supplementary material for: Limited Effect of Chronic Valproic Acid Treatment in a Mouse Model of Machado-Joseph Disease
Source: PLoS One. 2015 Oct 27;10(10):e0141610. doi: 10.1371/journal.pone.0141610 (PMC4624233; doi:10.1371/journal.pone.0141610)

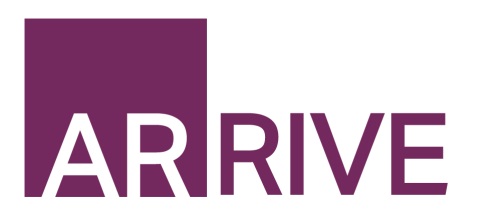


The ARRIVE Guidelines Checklist

Animal Research: Reporting In Vivo Experiments

Carol Kilkenny^1^, William J Browne^2^, Innes C Cuthill^3^, Michael Emerson^4^ and Douglas G Altman^5^

*^1^The National Centre for the Replacement, Refinement and Reduction of Animals in Research, London, UK, ^2^School of Veterinary Science, University of Bristol, Bristol, UK, ^3^School of Biological Sciences, University of Bristol, Bristol, UK, ^4^National Heart and Lung Institute, Imperial College London, UK, ^5^Centre for Statistics in Medicine, University of Oxford, Oxford, UK.*

|  | | ITEM | RECOMMENDATION | Section/ Paragraph |
| --- | --- | --- | --- | --- |
| 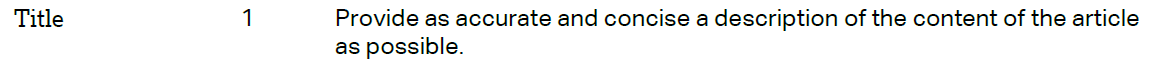 | | | Page 1 |  |
| 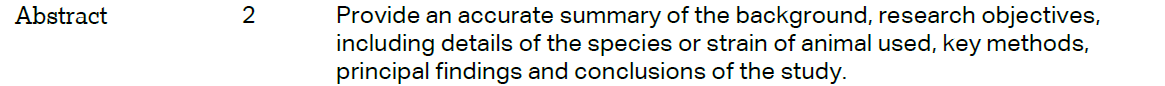 | | | Section “Abstract” Page 2 |  |
| INTRODUCTION | | |  |  |
| 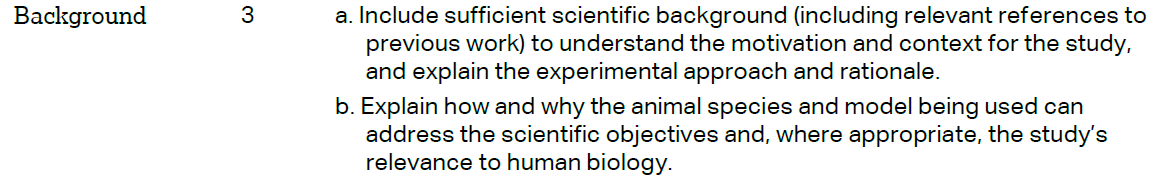 | | | **a.** Section “Introduction”  Pages 3-5  **b.** Section “Material and Methods” page 11, lines 5-6 |  |
| 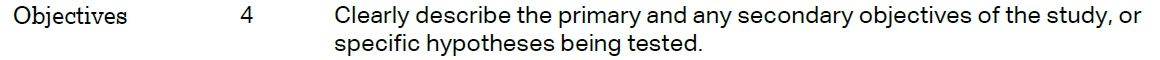 | | | Section “Introduction  Page 5, lines 1-2 |  |
| METHODS | | |  |  |
| 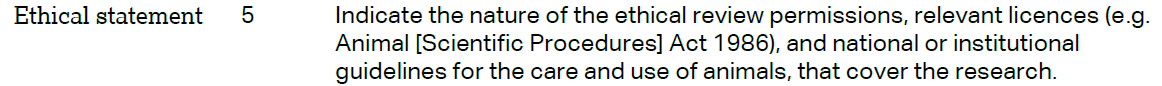 | | | Section “Material and methods”  Page 12, lines 2-11 |  |
| 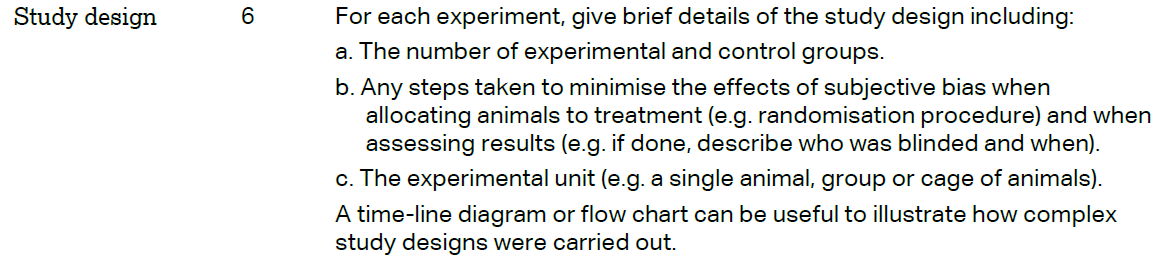 | | | **a.** Section “Material and Methods”, Sub-section “Behavioural Analyses” Page 12, lines 2-3  **b.** Section “Material and Methods”, Sub-section “Transgenic mice model and drug administration”, Page 12, lines 5-7  c. Section “Material and Methods”, Sub-section “Statistical analysis”.  Page 15  A timeline diagram can be found on Fig.1 |  |
| 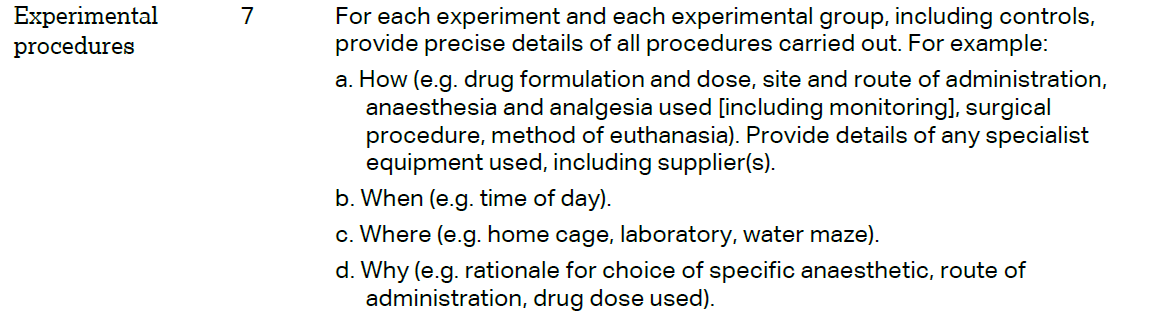 | | | Section “Material and Methods”, Sub-section “Transgenic mice model and drug administration”, Page 11 and 12  A time-line diagram can be found on Fig.1 |  |
| 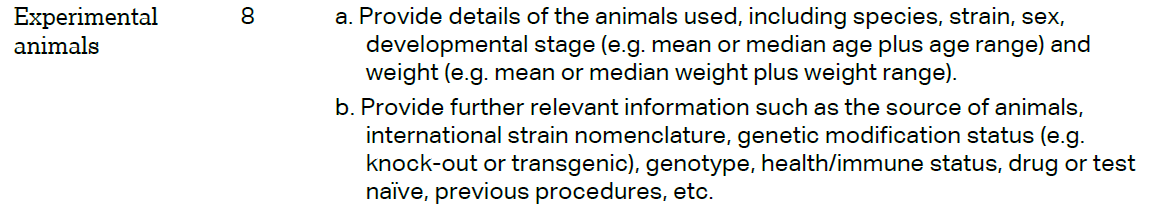 | | | Section “Material and Methods”, Sub-section “Transgenic mice model and drug administration”, Page 11 and 12  A time-line diagram can be found on Fig.1 |  |

The ARRIVE guidelines. Originally published in *PLoS Biology*, June 2010^1^

| 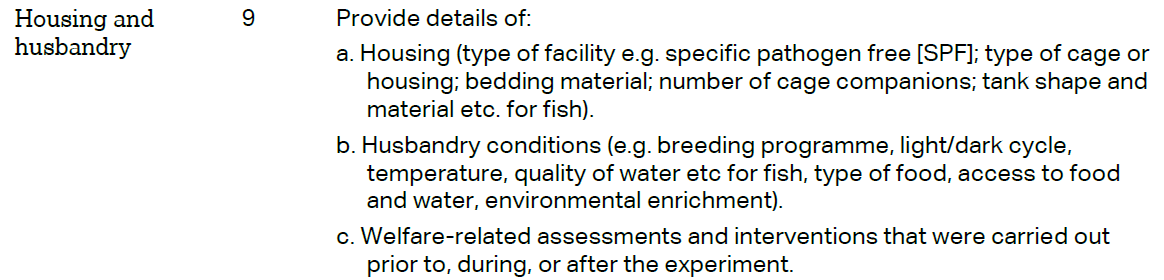 | Section “Material and Methods”, Sub-section “Transgenic mice model and drug administration”, Page 11 and 12  A time-line diagram can be found on Fig.1 | |
| --- | --- | --- |
| 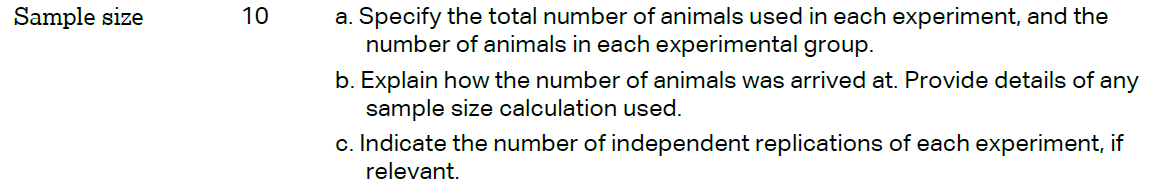 | Section “Material and Methods”, Sub-section “Statistical analysis”.  Page 15 | |
| 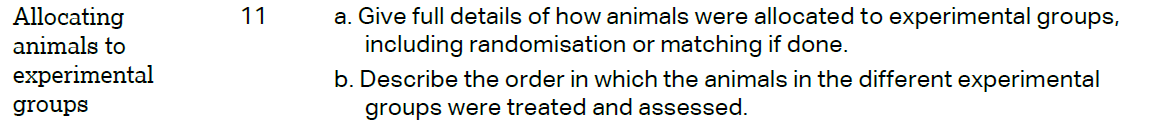 | Section “Material and Methods”, Sub-section “Transgenic mice model and drug administration”, Page 11 and 12 | |
| 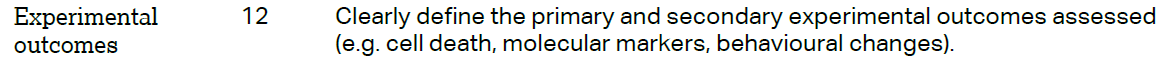 | Section “body weight”, “behavioural tests” and “Western-blot” of the section “Material and methods”  Page 18-20 | |
| 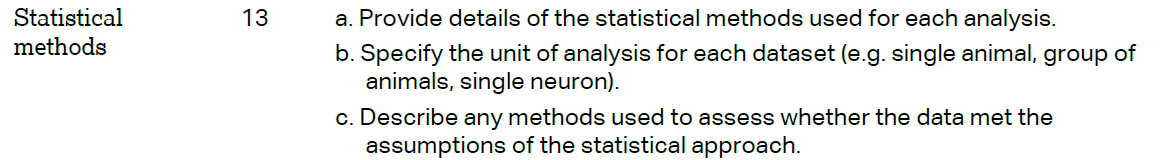 | Section “Material and Methods”, Sub-section “Statistical analysis”.  Page 15 | |
| RESULTS |  | |
| 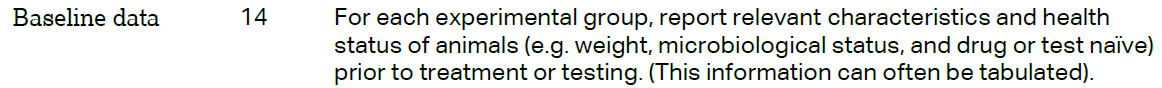 | First paragraph of the “Results” section (last sentence of the paragraph) | |
| 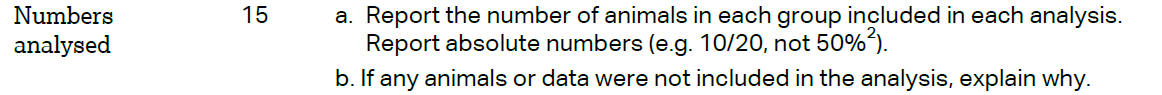 | The numbers of animals analysed can be found always in the legends of the figures. | |
| 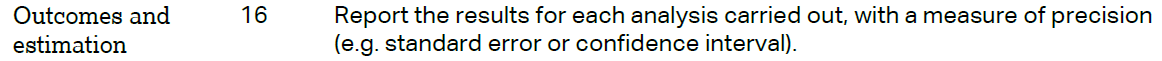 | Measures of precision (standard error mean) are found in the figures. | |
| 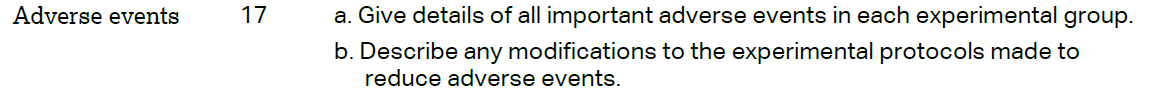 | The adverse events are discriminated in the section of the results “Survival and general health”  Page 9 | |
| DISCUSSION |  | |
| 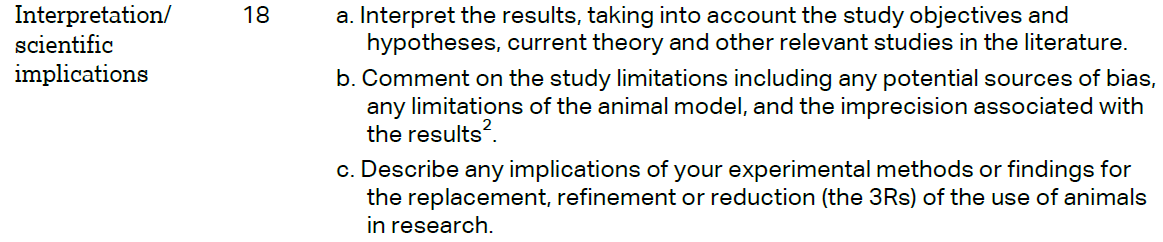 | Section “Discussion”  Page 14-16 | |
| 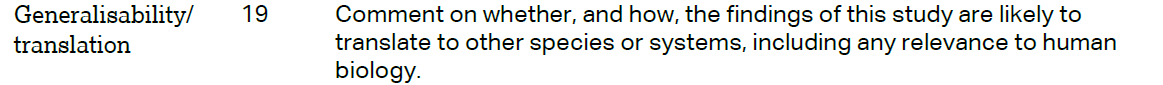 | Section “Discussion”, last paragraph of pages 14 and 16. | |
| 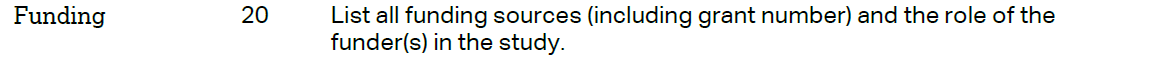 | | The funding details can be found on the online section “Financial Disclosure” and can be found on the first page of the pdf (non-numbered pages) |


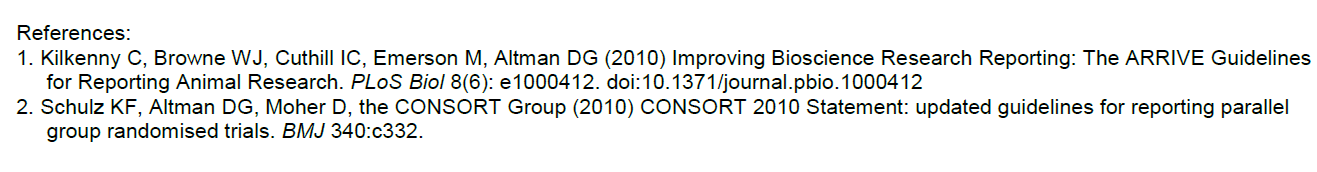

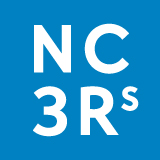

Supplement: S1 Checklist — (DOCX) [file pone.0141610.s001.docx]
