# Supplementary figures and images for: Limited Effect of Chronic Valproic Acid Treatment in a Mouse Model of Machado-Joseph Disease
Source: PLoS One. 2015 Oct 27;10(10):e0141610. doi: 10.1371/journal.pone.0141610 (PMC4624233; doi:10.1371/journal.pone.0141610)

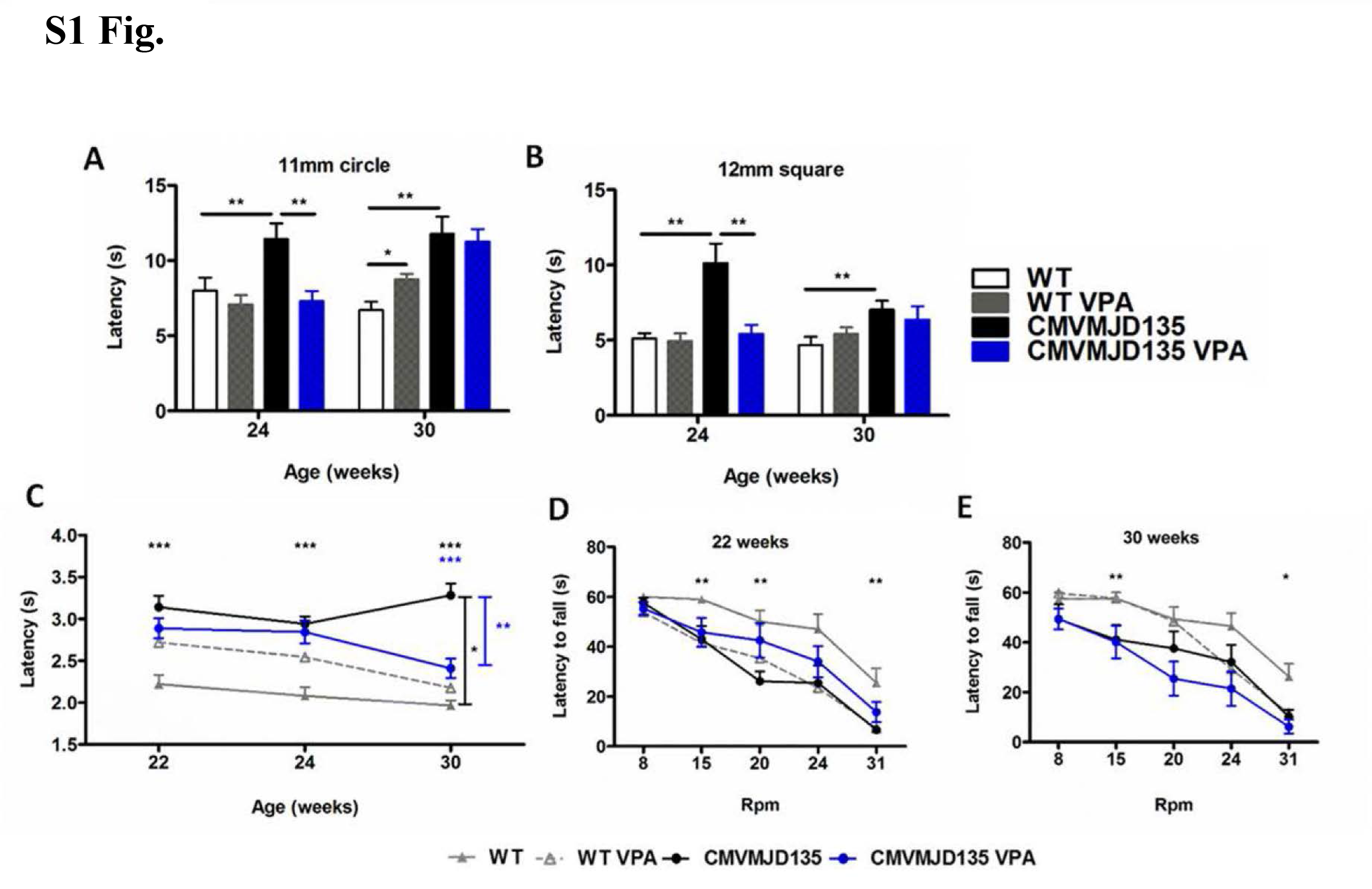

Supplement: S1 Fig — (A) Amelioration of balance and motor coordination at 24 weeks of age in 11 mm circle and (B) 12 mm square beams in beam walk test; (C) Motor swimming coordination improvement; (D,E) no improvement in increasing rotations in Rotarod were observed between VPA-treated and non-treated CMVMJD135. Bars represent the mean ± SEM (WT veh, n = 10; WT VPA, n = 15, CMVMJD135 vehicle, n = 10, CMVMJD135 VPA, n = 13), * represent p<0.05, ** represent p<0.01 and *** represent p<0.001, black asterisks represents the difference between WT and CMVMJD135, blue asterisks represents the difference between non-treated and VPA-treated CMVMJD135, (Repeated-measures ANOVA, Tukey correction for continuous variables, One-Way ANOVA for differences between groups in specific ages of the continuous variables and Mann-Whitney U test for continuous variables without normal distribution (Rotarod)). (TIF) [file pone.0141610.s002.tif]
